# Research progress on metabolic abnormalities in myocardial hypertrophy

**DOI:** 10.3389/fcvm.2026.1912835

**Published:** 2026-07-31

**Authors:** Xueru Liu, Yanlin Liu, Meng Huang, Fan Ouyang, Yukun Li

**Affiliations:** 1 Tumor ImmunoMetabolism Institute (TIMI), Zhuzhou Hospital Affiliated to Xiangya School of Medicine, Central South University, Zhuzhou, Hunan, China; 2 Shaoyang Central Hospital, Shaoyang, Hunan, China; 3 Department of Cardiology, the First Affiliated Hospital of USTC, Division of Life Sciences and Medicine, University of Science and Technology of China, Hefei, Anhui, China

**Keywords:** biomarkers, branched-chain amino acids, cardiac metabolism, fatty acid oxidation, glycolysis, ketone bodies, metabolic remodeling, metabolomics

## Abstract

Myocardial hypertrophy is initially an adaptive response to mechanical, neurohumoral, or metabolic stress, but persistent hypertrophy increases the risk of heart failure, arrhythmia, and death. Metabolic remodeling is now recognized as an early driver rather than a passive consequence of increased workload. This review aims to summarize recent advances in metabolic abnormalities in myocardial hypertrophy, focusing on pathophysiological mechanisms, biomarker implications, therapeutic opportunities, and future directions. Current evidence establishes that hypertrophic myocardium exhibits substrate inflexibility, impaired fatty acid oxidation, increased glycolysis with anaplerotic rerouting, mitochondrial calcium and quality-control defects, NAD^+^-sirtuin disruption, redox stress and ferroptosis, branched-chain amino acid accumulation, and ketone body adaptation. These abnormalities interact with canonical growth pathways including AMPK, mTOR, PKA, YAP, STAT3, ERR, SIRT3/SIRT5/SIRT6, and inflammatory programs. In biomarker research, metabolomics has moved from single metabolites toward integrated panels combining circulating acylcarnitines, amino acids, ketones, redox markers, and imaging-derived hypertrophy; however, interpretation remains strongly context-dependent on etiology, disease stage, sex, renal function, diabetes, and therapy. Therapeutically, phenotype-specific strategies—such as NAD^+^ repletion, SIRT activation, BCAA catabolism modulation, and ferroptosis inhibition—are emerging but require biomarker-guided trials. Despite these advances, critical challenges remain: distinguishing adaptive compensation from maladaptive remodeling, validating tissue-to-plasma concordance, establishing longitudinal human cohorts with serial metabolomics, and developing harmonized multi-omics pipelines. By addressing these core issues, this review provides a comprehensive, clinically oriented perspective on the current state and future trajectory of metabolic research in myocardial hypertrophy, emphasizing the need for biomarker-enriched interventions before irreversible remodeling occurs.

## 1 Introduction

Myocardial hypertrophy denotes an increase in cardiomyocyte size and ventricular mass that develops when the heart is exposed to sustained mechanical, neurohumoral, genetic, ischemic, inflammatory, or metabolic stress. Physiological hypertrophy, such as that induced by endurance training or pregnancy, usually preserves chamber function and metabolic flexibility. Pathological hypertrophy, in contrast, is commonly linked to hypertension, valvular disease, diabetes, obesity, cardiomyopathy, renal disease, or aging and may progress to fibrosis, capillary rarefaction, diastolic dysfunction, systolic failure, arrhythmia, and sudden death (1–3).

The adult heart has high ATP demand and normally oxidizes multiple substrates, especially fatty acids, glucose, lactate, ketone bodies, and amino acids. Hypertrophic growth challenges this flexible network. The heart must provide ATP for contraction, reducing equivalents for mitochondrial respiration, carbon skeletons for biosynthesis, redox buffering, and signals that coordinate cell growth. Foundational work established that metabolic remodeling is inseparable from contractile performance, growth, and survival (1, 4, 5). More recent studies show that metabolic changes can actively direct hypertrophic signaling, epigenetic state, immune recruitment, and cell death (6–10).

The topic is also increasingly relevant to biomarker research. Classical biomarkers such as natriuretic peptides and troponins reflect wall stress and injury but do not fully resolve the metabolic phenotype of hypertrophic myocardium. Metabolomics and lipidomics can identify substrate stress, mitochondrial bottlenecks, amino acid imbalance, redox dysregulation, and systemic drivers such as diabetes or chronic kidney disease (11–13). The central challenge is how to separate adaptive metabolic compensation from maladaptive remodeling and to translate mechanistic signals into clinically interpretable biomarker panels.

This review synthesizes recent evidence on key metabolic derangements—substrate inflexibility, mitochondrial dysfunction, BCAA accumulation, and ketone adaptation—in myocardial hypertrophy, and critically examines their potential as clinically interpretable biomarker modules and therapeutic targets, while highlighting the persistent challenges in distinguishing adaptive from maladaptive remodeling and translating pathway signals into practice.

## 2 Metabolic remodeling is a driver of hypertrophic growth

Hypertrophic myocardium does not simply consume more fuel; it reorganizes the routes by which carbon, electrons, and nitrogen move through the cell. In pressure-overload models, cardiomyocyte acetyl CoA carboxylase 2 deletion preserved fatty acid oxidation and prevented metabolic remodeling, showing that altered substrate handling can modify hypertrophic progression (4). Conversely, glucose carbon can be redirected toward aspartate biosynthesis to support nucleotide production and growth during hypertrophy (6). The accompanying editorial framed this as a shift from the question “which fuel makes ATP?” to “which metabolic route supports growth?” (14).

Several pathways now connect metabolic flux to canonical hypertrophic signaling. YAP can support compensatory hypertrophy by promoting aerobic glycolysis in pressure overload (8). The KLF7/PFKL/ACADL axis coordinates glycolytic and fatty acid programs during hypertrophy in male mice (15). PKA has also been positioned as a master regulator that distinguishes physiological from pathological hypertrophic growth (16). PRL2 directly dephosphorylates AMPKalpha2 and promotes cardiac hypertrophy, while OTUD1 deubiquitinates AMPKalpha2 and worsens diabetic cardiomyopathy with mitochondrial dysfunction (17, 18). These studies suggest that metabolic abnormalities are not downstream noise; they are embedded in the signaling logic of hypertrophic remodeling.

The stage of hypertrophy strongly modifies metabolic interpretation. Early adaptive hypertrophy may increase glucose uptake, amino acid use, and biosynthetic flux to support growth, while maintaining enough mitochondrial reserve to preserve contractile function. Later maladaptive hypertrophy often shows substrate inflexibility, mitochondrial dysfunction, oxidative stress, impaired calcium handling, and fibrosis. A metabolite that appears harmful in advanced disease may therefore be adaptive during early compensation. Lactate, ketones, and anaplerotic intermediates illustrate this problem: they can serve as fuels, redox buffers, or stress signals depending on stage, oxygen supply, insulin signaling, and mitochondrial capacity (6–8, 19).

**Figure 1:**
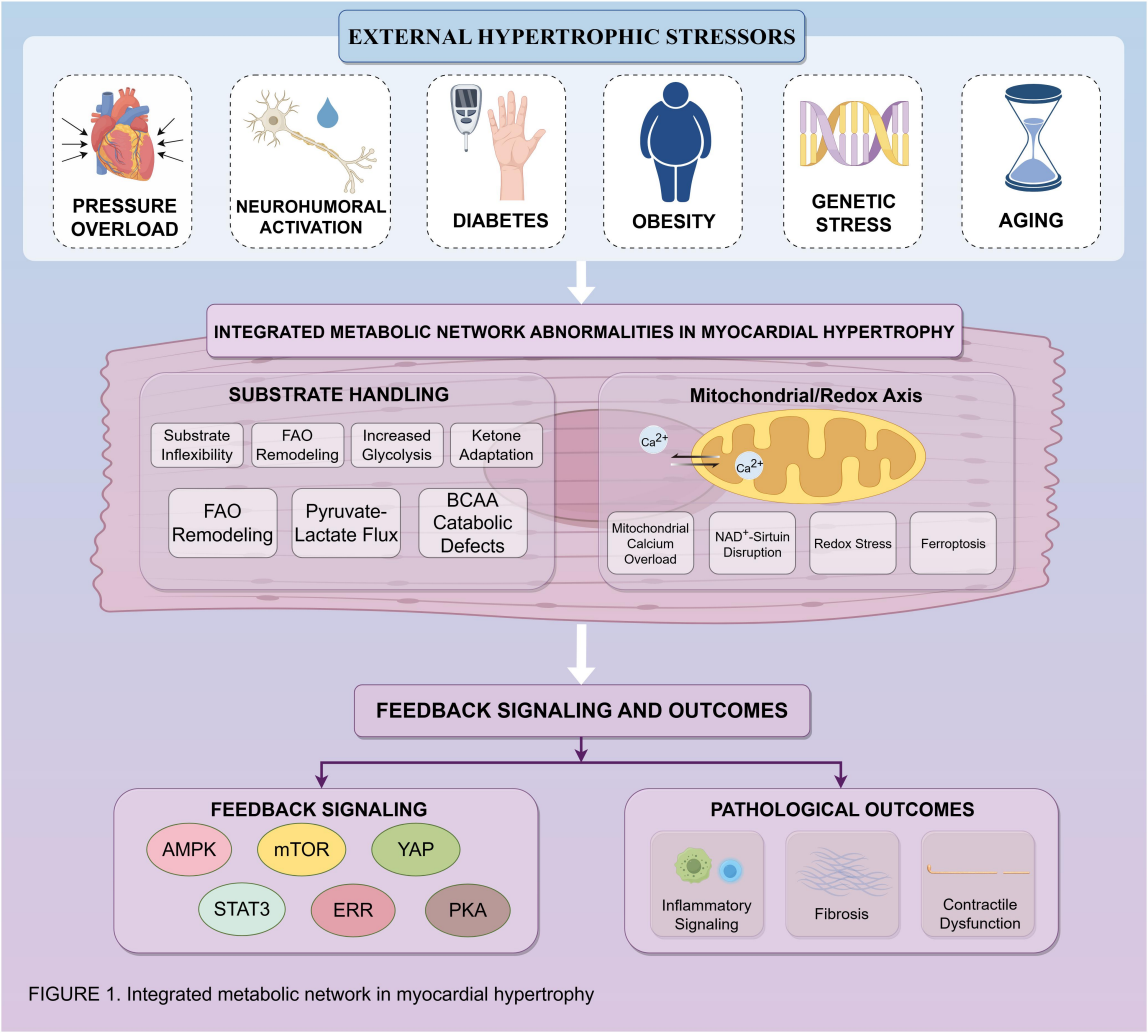
Integrated metabolic network in myocardial hypertrophy. Pressure overload, neurohumoral activation, diabetes, obesity, genetic stress, and aging converge on substrate inflexibility, FAO remodeling, increased glycolysis, pyruvate-lactate flux, mitochondrial calcium overload, NAD^+^-sirtuin disruption, redox stress, BCAA catabolic defects, ketone adaptation, and ferroptosis. These processes feed back into AMPK, mTOR, YAP, STAT3, ERR, PKA, inflammatory signaling, fibrosis, and contractile dysfunction.

Physiological and pathological hypertrophy also differ metabolically. Exercise-induced growth usually preserves or improves mitochondrial quality, vascular supply, and energetic reserve. Pressure overload, diabetes, obesity, and aging more often create a mismatch between cardiac growth and metabolic infrastructure. The regression literature emphasizes that hypertrophy can reverse when stress is removed, but metabolic recovery is not automatic (3). A Biomarker Research-oriented framework should therefore distinguish markers of growth, markers of energetic strain, markers of irreversible remodeling, and markers of therapeutic reversibility. The integrated metabolic network and major biomarker axes are summarized in Figure 1 and Table 1.

**Table 1:** Major metabolic abnormalities in myocardial hypertrophy and biomarker implications.

Metabolic axis	Typical abnormality in hypertrophy	Representative evidence	Candidate measurable readouts	Translational implication
Fatty acid uptake and oxidation	Reduced FAO reserve in pressure overload; lipotoxicity when lipid supply exceeds oxidation	ACC2 deletion altered pressure-overload remodeling; CD36 and diabetic lipotoxicity studies define uptake and lipid stress (4, 20, 25–28)	Free fatty acids, long-chain acylcarnitines, ceramides, triglyceride species, CD36-linked signatures	Interpret with diabetes, obesity, insulin resistance, and oxygen demand; both low and excessive FAO can be maladaptive
Glucose and glycolysis	Increased uptake, glycolytic rerouting, and biosynthetic anaplerosis	Glucose-to-aspartate biosynthesis, YAP-driven glycolysis, pyruvate-lactate axis, PANX1-glycolysis link (6–9)	Glucose, lactate, pyruvate, alanine, lactate/pyruvate ratio, glycolytic enzyme signatures	May identify growth-supporting metabolism before irreversible failure
Lactate and epigenetic lactylation	Lactate acts as fuel, redox carrier, and chromatin-linked signal	Myosin lactylation and histone lactylation studies link lactate to function and hypertrophy (10, 32)	Lactate, pyruvate, lactylation-related protein or chromatin markers in tissue studies	Distinguish adaptive lactate oxidation from maladaptive lactylation programs
Mitochondrial quality and calcium	Impaired oxidative phosphorylation, fission-fusion imbalance, mitophagy defects, calcium overload	DYRK1B-STAT3, Bmal1, MICU3, FARS2, FMO2, and mitochondrial stabilization studies (36–41, 43)	Acylcarnitines, TCA intermediates, mitochondrial DNA, redox markers, imaging of energetics where available	Central node for risk stratification and therapeutic monitoring
NAD^+^-sirtuin-redox network	NAD^+^ depletion, altered deacylation, oxidative stress, ferroptosis	NAD^+^ repletion, nicotinamide, SIRT3/SIRT5/SIRT6, alpha-ketoglutarate, and CAV1-ferroptosis data (21, 27, 45–48, 51)	NAD-related metabolites, glutathione markers, lipid peroxidation products, iron-linked markers	Candidate response markers for NAD^+^, sirtuin, and ferroptosis-targeted therapies
BCAA metabolism	BCAA accumulation or impaired catabolism in heart failure, diabetes, and HFpEF	Defective catabolism promotes heart failure; newer BCAA-targeted and GRSF1 studies extend therapeutic relevance (53–60)	Leucine, isoleucine, valine, branched-chain ketoacids, C3/C5 acylcarnitines	Strong cardiometabolic biomarker axis, but requires systemic context
Ketone bodies	Increased ketone use or intrinsic ketogenesis as adaptive stress response	Ketone-heart reviews and intrinsic ketogenic capacity in NAD^+^ therapy (19, 63, 64)	Beta-hydroxybutyrate, acetoacetate, acetylcarnitine, NAD-related markers	Interpretation depends on fasting, diabetes, SGLT2 inhibitor use, diet, and renal function

Sex, age, and comorbidity should not be treated as nuisance variables. The KLF7/PFKL/ACADL axis was reported in male mice, which highlights that sex-specific metabolic regulation may influence translation (15). Diabetes changes lipid supply, glucose utilization, mitochondrial redox state, and ferroptosis susceptibility (20–22). Kidney disease alters amino acids, acylcarnitines, and uremic metabolites. Diet, fasting state, exercise, and medications such as SGLT2 inhibitors change ketones and substrate preference. These factors should be recorded systematically in metabolomic studies, not adjusted away after the fact.

## 3 Pathophysiological mechanisms: metabolic inflexibility and organelle dysfunction in hypertrophic hearts

### 3.1 Substrate metabolism: fatty acids, glucose, and lactate

The healthy adult heart derives much of its ATP from fatty acid oxidation (FAO), but pathological hypertrophy often reduces FAO capacity and increases reliance on glucose and lactate. This fetal-like pattern may initially improve oxygen efficiency, yet chronic substrate inflexibility can lower ATP reserve, promote lipid intermediate accumulation, and intensify oxidative stress (1, 5, 23, 24). CD36, a fatty acid transporter and metabolic signaling node, is central to myocardial lipid uptake and cardiometabolic disease (25). In diabetic states, excessive lipid supply and impaired mitochondrial handling can produce lipotoxicity, ceramide accumulation, contractile dysfunction, and ferroptosis-like injury (20, 21, 26–29).

Recent work supports both sides of the FAO question. In some hypertrophic and failing contexts, inadequate FAO contributes to energetic failure, and pan-ERR agonists that enhance cardiac fatty acid metabolism and mitochondrial function improved heart failure phenotypes (30). In diabetic cardiomyopathy, however, excessive or poorly matched mitochondrial FAO may also be harmful; targeting Decr1 improved cardiomyopathy by suppressing mitochondrial FAO in diabetic mice (28). Therefore, FAO biomarkers should not be interpreted as simply “high is bad” or “low is bad.” Acylcarnitine patterns, free fatty acids, lipid species, insulin resistance, oxygen delivery, and hypertrophy etiology must be interpreted together.

### 3.2 Glucose uptake, glycolysis, pyruvate-lactate flux, and anaplerosis

Increased glucose uptake and glycolysis are among the most reproducible features of hypertrophic remodeling, but the fate of glucose carbon varies. It can support ATP generation, replenish tricarboxylic acid cycle intermediates, generate biosynthetic precursors, or feed lactate and epigenetic pathways (6–10). The pyruvate-lactate axis has emerged as a regulatory module in cardiac hypertrophy and heart failure, linking cytosolic redox state, mitochondrial oxidation, lactate handling, and growth signaling (7). Cardiomyocyte PANX1 was recently shown to control glycolysis and neutrophil recruitment in hypertrophy, connecting metabolic flux with inflammation (9).

Glucose carbon also supplies non-energetic pathways that matter for hypertrophy. The pentose phosphate pathway supports nucleotide synthesis and redox buffering. The hexosamine biosynthetic pathway links nutrient excess to protein modification. Serine and one-carbon metabolism can connect growth, methylation, and antioxidant capacity. Anaplerotic entry into the tricarboxylic acid cycle supports biosynthesis but can also signal mitochondrial congestion if oxidative capacity is limited. These routes are difficult to infer from glucose uptake alone, which is why tracer studies and integrated metabolomics remain important (11, 12, 31).

Lactate deserves particular caution. It is no longer reasonable to treat lactate simply as evidence of anaerobic failure. The hypertrophic heart can use lactate as fuel, export lactate to manage cytosolic redox state, or use lactate-derived signals to influence gene regulation. Lactylation and other metabolite-linked epigenetic mechanisms provide a plausible bridge between transient metabolic changes and durable hypertrophic programs (10). Clinically, this means that blood lactate is too crude for myocardial interpretation, but lactate-pyruvate ratios, myocardial imaging, and tissue or plasma multi-omics may reveal more specific information about redox pressure and metabolic rerouting.

Lactate is no longer viewed only as a waste product or emergency fuel. Lactate-derived histone lactylation can influence gene expression, and recent studies connect lactylation to cardiac structure and function. Alpha-myosin heavy chain lactylation maintained sarcomeric structure and mitigated heart failure development (32), whereas lactate-regulated histone lactylation was implicated in pathological hypertrophy (10). These findings point to lactate as a context-dependent signal: it may support adaptive remodeling when coupled to mitochondrial oxidation and sarcomeric maintenance, but it may also amplify maladaptive transcription when lactate production, clearance, or chromatin regulation becomes uncoupled. Beyond histones, direct lactylation of glycolytic enzymes such as enolase 1 (ENO1) can create a feed-forward loop that amplifies lactate synthesis and epigenetic reprogramming, offering a potential paradigm for self-sustaining metabolic remodeling in hypertrophy (33). Beyond metabolic enzymes, recent evidence identifies protein disulfide isomerase (P4HB) as a novel lactylation target; its K311-site lactylation exacerbates cardiac injury by disrupting mitochondrial function and kynurenine metabolism, which may also contribute to hypertrophic remodeling (34). Lactylation is regulated by a conserved writer–eraser–reader machinery, which may also operate in cardiac hypertrophy (35).

### 3.3 Mitochondrial bioenergetics, dynamics, calcium, and quality control

Mitochondria sit at the center of hypertrophic metabolism. They integrate substrate oxidation, ATP synthesis, reactive oxygen species, calcium signaling, cell death pathways, and innate immune activation. DYRK1B-STAT3 signaling can drive hypertrophy and heart failure by impairing mitochondrial bioenergetics (36). Mitochondrial calcium overload contributes to diabetic cardiomyopathy, as shown by Bmal1 downregulation and Bcl2/IP3R-mediated calcium handling (37). MICU3 directly regulates mitochondrial calcium and cardiac hypertrophy (38), and FMO2 appears to protect against pathological hypertrophy by maintaining endoplasmic reticulum-mitochondria association through the IP3R2-Grp75-VDAC1 complex (39).

Mitochondrial morphology and quality control are equally important. Drp1-dependent fission, mitophagy, and mitochondrial homeostasis shape the transition from compensated hypertrophy to failure (40–43). Mitophagy was required to maintain function during high-fat diet-induced diabetic cardiomyopathy (42). FARS2 deficiency caused cardiomyopathy by disrupting mitochondrial homeostasis and quality control (41). Small molecules or natural products that stabilize mitochondrial integrity, including SIRT3 activation and USP9X/MCL1-mediated mitochondrial stabilization, reduced hypertrophy or heart failure phenotypes in experimental studies (44, 45).

### 3.4 NAD^+^, sirtuins, redox stress, and ferroptosis

NAD^+^ biology links energy metabolism, redox state, DNA repair, mitochondrial signaling, and protein deacylation. In HFpEF models, NAD^+^ repletion reversed disease features and nicotinamide improved HFpEF-related phenotypes (46, 47). SIRT6 mitigated diabetic HFpEF (48), SIRT5 improved fatty acid metabolism through CPT2 desuccinylation in diabetic cardiomyopathy (27), and SIRT3 activation alleviated myocardial hypertrophy and fibrosis by restoring mitochondrial homeostasis (45). Canagliflozin also preserved SIRT3 expression while improving mitochondrial metabolism in salt-induced hypertrophy (49).

The NAD^+^/NADH redox balance serves as a primary rheostat for dehydrogenase-catalyzed steps, dictating the throughput of substrate catabolism. Its depression in failing myocardium curtails oxidative fuel utilization and fosters energetic derangement (50). Redox imbalance may operate upstream and downstream of metabolic remodeling. Oxidative stress pathways are prominent in diabetic cardiomyopathy (22), and ferroptosis-related injury has been connected to cardiomyopathy and heart failure phenotypes (21, 51). Alpha-ketoglutarate improved cardiac insufficiency through NAD^+^-SIRT1 signaling, mitophagy, and ferroptosis regulation in pressure overload (51). These data support biomarker panels that include redox-sensitive metabolites, iron handling, glutathione-linked readouts, NAD-related metabolites, and mitochondrial injury signals rather than isolated oxidative stress markers. Of note, the NF-B/HSPB1 axis has recently emerged as a modulator of ferroptosis in other disease settings, suggesting potential parallels in the hypertrophic heart (52).

### 3.5 Branched-Chain amino acids and nitrogen-carbon coupling

Branched-chain amino acids (BCAAs) are among the strongest metabolomic signals in cardiometabolic disease. Early mechanistic evidence showed that defective BCAA catabolism promotes heart failure (53). Subsequent work linked mitochondrial H2S to BCAA catabolism (54), showed that extra-cardiac BCAA catabolism can lower blood pressure and protect from heart failure (55), and summarized BCAAs as cardiovascular disease biomarkers and mediators (56). Circulating BCAA concentrations also associate with cardiometabolic disease risk (57).

The newest data move BCAAs from association to therapeutic targeting. Impaired cardiac BCAA metabolism was reported in a diabetic cardiomyopathy model (58). BCAA catabolism-targeted therapy improved HFpEF phenotypes (59), and GRSF1 protected against heart failure by maintaining BCAA homeostasis (60). These findings make BCAA pathway markers attractive for myocardial hypertrophy, especially when hypertrophy coexists with obesity, diabetes, hypertension, or kidney disease. The key enzymes BCAT, BCKDH, and BCKDK, along with mTOR and MEK-ERK signaling, are central to BCAA metabolic regulation in disease contexts (61). Still, circulating BCAA levels reflect skeletal muscle, liver, adipose tissue, kidney function, diet, microbiome metabolism, and drug exposure. A useful biomarker strategy should therefore pair plasma BCAAs with downstream ketoacids, acylcarnitines, insulin resistance, renal function, and cardiac imaging. Gut-derived metabolites may add another layer; indole-3-propionic acid has been reported to protect against HFpEF, supporting microbiome-host metabolic axes as candidate modifiers of hypertrophic remodeling (62).

### 3.6 Ketone bodies and alternative fuel adaptation

Ketone-body metabolism has become a major theme in cardiac energetics. Ketones can act as fuels, redox modulators, histone deacetylase-related signals, and mitochondrial stress modifiers (19, 63). In failing or hypertrophic myocardium, increased ketone oxidation may represent adaptive fuel selection when fatty acid and glucose pathways are inefficient. The heart also has intrinsic ketogenic capacity that appears to mediate NAD^+^ therapy in HFpEF (64).

From a biomarker perspective, beta-hydroxybutyrate and acetoacetate should be interpreted with caution. They may indicate fasting, diabetes, SGLT2 inhibitor exposure, ketogenic diet, renal function, or myocardial fuel preference. Their value may be greatest when integrated with NAD^+^ markers, acylcarnitines, lactate/pyruvate ratios, insulin resistance, and treatment context.

### 3.7 Diabetes, HFpEF, and inter-organ metabolic crosstalk

Diabetes and obesity create a systemic metabolic environment that accelerates hypertrophy and heart failure. Mechanisms include insulin resistance, lipotoxicity, microvascular dysfunction, inflammation, mitochondrial dysfunction, oxidative stress, altered adipokines, renal-cardio-metabolic crosstalk, and impaired substrate switching (22, 26, 29, 65). Human HFpEF metabolomics revealed myocardial metabolic signatures that differ from non-failing myocardium and reflect both cardiac and systemic disease biology (12). Integrated human cardiac metabolism studies reinforce that the failing heart cannot be understood without considering substrate delivery, oxygen use, organ crosstalk, and patient phenotype (13, 31).

Recent diabetic cardiomyopathy studies sharpen this view. Metabolic coordination structures contributed to diabetic myocardial dysfunction (66). CAV1 inhibition reduced ferroptosis through NRF2/GCLC signaling (21). OTUD1 drove diabetic cardiomyopathy by deubiquitinating AMPKalpha2 and inducing mitochondrial dysfunction (18). These studies suggest that hypertrophy in diabetes is not only a myocardial response to load; it is a multi-organ metabolic disease that converges on the myocardium (67).

HFpEF is a useful test case because it rarely arises from one metabolic abnormality. Obesity, hypertension, diabetes, renal dysfunction, aging, inflammation, skeletal muscle dysfunction, and endothelial impairment converge on concentric remodeling and exercise intolerance. NAD^+^ depletion, BCAA catabolic defects, ketone adaptation, and microbiome-linked metabolites each capture part of this systemic state (46, 47, 59, 62, 65). The question is not which single pathway defines HFpEF, but whether pathway modules can identify treatable subphenotypes. For example, one patient may have dominant insulin resistance and lipotoxicity, another may have BCAA catabolic impairment and renal dysfunction, and another may have mitochondrial redox stress with preserved glucose handling. Representative recent open-access studies informing these modules are summarized in Table 2.

**Table 2:** Representative recent open-access studies informing the field.

Ref	Year	Model or setting	Main metabolic finding	Relevance to myocardial hypertrophy
(6)	2020	Pressure-overload hypertrophy	Glucose carbon redirected to aspartate biosynthesis	Shows growth-supporting anaplerosis rather than simple ATP substitution
(7)	2021	Hypertrophy and heart failure models	Pyruvate-lactate axis modulates hypertrophy and failure	Connects lactate handling with remodeling severity
(47)	2021	HFpEF models	NAD^+^ repletion reversed HFpEF features	Supports NAD-related biomarkers and metabolic therapy
(8)	2022	Pressure overload	YAP mediated compensatory hypertrophy through aerobic glycolysis	Defines glycolysis as adaptive in selected stages
(48)	2022	Diabetic HFpEF	SIRT6 mitigated diabetic HFpEF	Links deacetylation, metabolism, and hypertrophy-related HFpEF
(15)	2023	Cardiac hypertrophy in male mice	KLF7/PFKL/ACADL axis modulated metabolic remodeling	Integrates glycolytic and fatty acid pathways
(12)	2023	Human HFpEF myocardium	Myocardial metabolomics revealed disease-specific signatures	Provides human biomarker relevance
(30)	2024	Heart failure models	Pan-ERR agonists enhanced FAO and mitochondrial function	Demonstrates metabolic nuclear receptor therapy potential
(45)	2024	Myocardial hypertrophy and fibrosis	SIRT3 activator improved mitochondrial homeostasis	Supports mitochondrial-sirtuin targeting
(9)	2024	Hypertrophy models	Cardiomyocyte PANX1 controlled glycolysis and neutrophil recruitment	Links metabolism to inflammatory remodeling
(66)	2025	Diabetic myocardial dysfunction	Metabolic coordination structures contributed to dysfunction	Highlights spatial and inter-cellular metabolic organization
(59)	2025	HFpEF models	BCAA catabolism-targeted therapy improved disease features	Moves BCAA markers toward therapeutic stratification
(60)	2026	Heart failure	GRSF1 maintained BCAA homeostasis and protected the heart	Shows current momentum in amino acid homeostasis

Inter-organ crosstalk also complicates biomarker interpretation. Plasma metabolites are not cardiac-specific. Acylcarnitines can reflect skeletal muscle, liver, adipose tissue, kidney clearance, and mitochondrial stress. BCAAs are shaped by diet, microbiome, liver metabolism, insulin resistance, and muscle turnover. Ketones are influenced by fasting, diabetes, SGLT2 inhibitor use, and hepatic production. Gut-derived indoles and other microbial metabolites may affect vascular and myocardial biology but also reflect diet and intestinal ecology (62). A strong biomarker study must therefore pair circulating markers with cardiac phenotyping such as LV mass, strain, perfusion reserve, CMR tissue characterization, or myocardial tissue data when available.

## 4 Clinical implications of metabolic biomarkers

Metabolic biomarkers for myocardial hypertrophy should meet three standards. First, they should be biologically interpretable: a metabolite panel should map to a pathway such as FAO bottleneck, glycolytic rerouting, mitochondrial stress, BCAA catabolic defect, NAD^+^ depletion, ketone adaptation, or ferroptosis. Second, they should be clinically contextualized: a signature must be interpreted against hypertension, diabetes, kidney function, sex, age, diet, exercise, medications, and heart failure stage. Third, they should be actionable: the marker should identify risk, monitor regression, stratify therapy, or reveal a targetable metabolic liability. The rationale for pathway-anchored biomarker panels aligns with the emerging multiple-marker approach advocated for cardiovascular disease, which integrates traditional and novel markers to improve diagnostic accuracy and risk stratification (68).

Candidate circulating markers include long-chain acylcarnitines and lipid species for FAO mismatch; lactate, pyruvate, and alanine for cytosolic redox and glycolytic pressure; BCAAs and branched-chain ketoacids for amino acid catabolic stress; beta-hydroxybutyrate and acetoacetate for ketone adaptation; NAD-related metabolites for sirtuin-linked bioenergetics; alpha-ketoglutarate, succinate, citrate, and malate for tricarboxylic acid cycle state; and glutathione, iron-linked markers, or lipid peroxidation products for redox and ferroptosis. Tissue studies remain essential, but clinical translation will likely depend on plasma panels combined with imaging, natriuretic peptides, troponin, renal function, and machine-learning models that are externally validated (11–13).

Methodological rigor is decisive. Advances in NMR and UPLC-MS have established metabolomics as a high-throughput platform for quantitative characterization of metabolic profiles in cardiovascular research (69). Pre-analytic variation can dominate metabolomic signals: fasting duration, sample tube, processing delay, storage temperature, freeze-thaw cycles, hemolysis, exercise, and acute illness all change metabolite abundance. Targeted assays offer better reproducibility for clinical translation, while untargeted platforms are valuable for discovery but require batch correction, internal standards, and independent replication. Reports should specify whether metabolites were measured in plasma, serum, whole blood, tissue, or extracellular vesicles. They should also report medication exposure, renal function, diabetes status, diet, and time of day.

A second requirement is pathway anchoring. Integrated multi-omics approaches have revealed metabolic heterogeneity in hypertrophic cardiomyopathy, enabling subtype-specific risk stratification and therapeutic strategies (70). A statistical metabolite panel that predicts LV mass but cannot be mapped to a plausible pathway may be useful for risk prediction but weak for therapy selection. Conversely, a biologically elegant pathway marker may fail clinically if it adds no information beyond blood pressure, diabetes, kidney function, natriuretic peptides, and imaging. The best candidates will satisfy both conditions: they will report a pathway such as FAO bottleneck, BCAA catabolic defect, NAD^+^ depletion, redox stress, or ketone adaptation, and they will improve prediction or treatment response beyond standard variables.

The third requirement is longitudinal validation. Molecular diagnostics encompassing metabolomics offer unprecedented insights into heart failure pathogenesis, aiding early diagnosis and personalized management (71). Cross-sectional metabolomics cannot distinguish cause, compensation, and consequence. A metabolite signature should be tested before hypertrophy develops, during progression, during regression after pressure unloading or risk-factor treatment, and in relation to clinical endpoints. Longitudinal study designs are essential to determine whether a metabolic marker changes prior to increases in LV mass, following hypertrophic remodeling, or only during advanced heart failure. They can also determine whether metabolic improvement precedes structural regression. This is essential if biomarkers are to guide earlier intervention rather than merely describe established disease.

## 5 Therapeutic opportunities and future directions

### 5.1 Therapeutic opportunities and cautions

Metabolic therapy for myocardial hypertrophy should be phenotype-specific. Potential strategies include restoring mitochondrial quality control, improving NAD^+^-sirtuin signaling, redirecting glycolysis and anaplerosis, normalizing BCAA catabolism, modulating ketone metabolism, correcting lipotoxicity, and reducing ferroptosis (27, 28, 30, 45–47, 49, 51, 59, 64). SGLT2 inhibitors, DPP-4 inhibitors, ERR agonism, SIRT activation, BCAA catabolic modulation, and mitochondrial stabilizers are all represented in recent experimental or translational literature (20, 28, 30, 45, 49, 59, 64). Newer pathway work also implicates histamine N-methyltransferase upregulation in cardiac hypertrophy and heart failure, broadening the metabolic map beyond classical energy substrates (72).

However, metabolic pathways are not universally beneficial or harmful. Increasing FAO may help one phenotype and worsen another (73). Lactate can be an adaptive fuel, a redox marker, or an epigenetic signal (10). Ketones can reflect therapeutic adaptation or uncontrolled metabolic stress (19). BCAA lowering may help patients with impaired catabolism but may not be appropriate in cachexia or advanced frailty (74). The efficacy of metabolic interventions thus depends critically on HF phenotype, disease stage, and global metabolic network integrity (75). Biomarker-guided trials are therefore needed to test whether metabolic signatures can select patients, define dose, and track response.

Future trials should use metabolic enrichment rather than broad unselected enrollment (76). A trial targeting BCAA catabolism should enroll patients with evidence of BCAA pathway dysfunction, not all patients with hypertrophy. A trial of NAD^+^ repletion should measure NAD-related metabolites, mitochondrial function, and sirtuin-linked pathway markers. A trial aimed at lipotoxicity should define lipid overload and mitochondrial FAO mismatch at baseline. Such designs would be smaller but more informative, because they test whether a pathway-defined phenotype responds to a pathway-matched intervention.

Safety and tradeoffs must be explicit. Suppressing fatty acid oxidation may reduce lipotoxic intermediates in diabetes but could impair ATP supply in a heart that remains dependent on fatty acids. Stimulating FAO may improve lipid handling but worsen oxygen efficiency. Ketone elevation may be adaptive in HFpEF but dangerous in uncontrolled diabetes. BCAA catabolic activation may benefit metabolic overload but could be inappropriate in malnutrition, sarcopenia, or cancer cachexia. Metabolic medicine in hypertrophy therefore needs the same precision logic as oncology: define the targetable liability, measure pathway engagement, and monitor both efficacy and toxicity (77).

**Figure 2:**
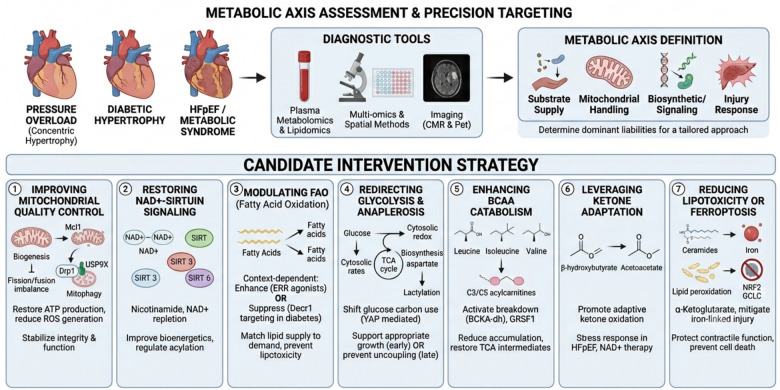
Therapeutic targeting of metabolic liabilities in myocardial hypertrophy. Candidate interventions include improving mitochondrial quality control, restoring NAD+-sirtuin signaling, modulating FAO, redirecting glycolysis and anaplerosis, enhancing BCAA catabolism, leveraging ketone adaptation, and reducing lipotoxicity or ferroptosis. The figure emphasize phenotype-specific selection rather than uniform metabolic activation or suppression.

Therapeutic targeting logic and practical biomarker-development steps are summarized in Figure 2 and Table 3.

**Table 3:** Practical framework for biomarker development.

Intended use	Candidate metabolic panel	Companion data needed	Main limitation	Next validation step
Early detection of maladaptive hypertrophy	Acylcarnitines, lactate/pyruvate, BCAAs, TCA intermediates, NAD-related metabolites	Blood pressure, LV mass, wall thickness, strain, natriuretic peptides	Subclinical hypertrophy is heterogeneous	Longitudinal cohorts before heart failure onset
Etiology stratification	Lipid species for diabetes/obesity, BCAA-ketoacid axis, ketones, redox markers	Diabetes status, renal function, diet, medications, sex, age	Strong systemic confounding	Multivariable models with external validation
Therapy selection	NAD^+^-sirtuin markers, FAO/acylcarnitine state, BCAA catabolic markers, ketone markers	SGLT2 inhibitor use, nutritional status, renal function, exercise capacity	Same pathway may be adaptive or maladaptive by stage	Biomarker-enriched interventional trials
Monitoring regression	Decline in maladaptive acylcarnitines or BCAAs, improved redox and mitochondrial markers	Serial imaging, fibrosis markers, symptoms, exercise testing	Plasma may not mirror myocardial tissue	Paired imaging-metabolomics follow-up
Mechanistic discovery	Untargeted metabolomics plus transcriptomics, proteomics, and spatial methods	Tissue source, ischemia time, comorbidity annotation	Batch effects and tissue-plasma discordance	Harmonized multi-center omics pipelines

Integration with imaging can make metabolic biomarkers clinically meaningful. LV mass and wall thickness define the structural phenotype; GLS and myocardial work report contractile reserve; CMR extracellular volume and T1 mapping report diffuse fibrosis; perfusion imaging reports microvascular reserve. Population-based studies have demonstrated that LV wall thickness and filling parameters are independently associated with circulating acylcarnitines and amino acid metabolites (78). In hypertrophic cardiomyopathy, CMR-derived wall thickness and tissue characterization have been integrated with myocardial metabolomics to reveal disease-specific metabolic signatures (79). When combined with metabolite modules, these measures can separate metabolically active but reversible hypertrophy from fibrotic, energetically depleted, or inflammatory disease. This integrated approach is likely to outperform either metabolomics or imaging alone, especially in HFpEF and diabetic hypertrophy where systemic confounding is strong.

### 5.2 Knowledge gaps

Several gaps limit translation. Longitudinal human data are sparse, especially before overt heart failure. A recent longitudinal lipidomic study in American Indians demonstrated that changes in circulating lipids over time were significantly associated with changes in left ventricular mass index (80). Sex-specific and ancestry-specific metabolic signatures remain underdeveloped. Tissue-to-plasma concordance is incompletely defined, and many animal studies use young male mice with single-stressor models. Standardized sample handling, fasting status, metabolomics platforms, medication annotation, renal function adjustment, and external validation are needed. Finally, multi-omics signatures should be linked to clinically meaningful outcomes such as hypertrophy regression, fibrosis progression, arrhythmia burden, exercise capacity, hospitalization, and mortality.

### 5.3 Disease-specific metabolic phenotypes

A clinically useful review must move beyond the idea that there is one metabolic program for all myocardial hypertrophy. In pressure overload, the dominant issue may be oxygen-efficient ATP production, capillary mismatch, mitochondrial stress, and transition from compensated growth to failure. In diabetic hypertrophy, lipotoxicity, insulin resistance, mitochondrial calcium overload, oxidative stress, and ferroptosis may dominate (20–22, 37, 72). In HFpEF, systemic inflammation, renal dysfunction, obesity, BCAA metabolism, NAD^+^ depletion, and endothelial dysfunction may shape the phenotype (46, 47, 59, 65). In genetic or sarcomeric hypertrophy, energetic inefficiency and impaired relaxation may be present even before overt remodeling. These phenotypes can share increased wall thickness while requiring different metabolic interpretation.

This distinction changes biomarker design. A lipidomic signature may be highly informative in obesity-related or diabetic hypertrophy but less central in pressure overload without metabolic syndrome. Obesity-related lipotoxicity drives ceramide accumulation, mitochondrial dysfunction, and inflammatory responses that accelerate hypertrophic remodeling (81). A ketone signature may reflect adaptive myocardial fuel selection in HFpEF, fasting state, SGLT2 inhibitor exposure, or uncontrolled diabetes. A BCAA signature may identify impaired amino acid catabolism in cardiometabolic disease, but in a frail patient it may also reflect malnutrition, skeletal muscle loss, or renal dysfunction. A NAD-related signal may identify mitochondrial and sirtuin-linked vulnerability, but it must be interpreted with age, inflammation, and renal handling. Therefore, biomarker panels should be built around clinical phenotype rather than applied uniformly.

A practical framework is to define four metabolic axes for each patient or cohort. The first is substrate supply: fatty acids, glucose, lactate, ketones, amino acids, and dietary context. Pressure overload drives complex shifts across glucose, fatty acid, branched-chain amino acid, and ketone body metabolism (82). The second is mitochondrial handling: oxidation, calcium, redox balance, mitophagy, fission-fusion, and electron transport capacity. The third is biosynthetic and signaling use: anaplerosis, amino acid signaling, protein acetylation, lactylation, succinylation, and kinase pathways such as AMPK, mTOR, PKA, and YAP. The fourth is injury response: ferroptosis, lipotoxicity, inflammatory recruitment, fibrosis, and cell death. This axis-based framework can organize complex multi-omics data into clinically interpretable modules.

### 5.4 From metabolite discovery to clinical decision support

Metabolomics is most valuable when it helps a decision. For myocardial hypertrophy, potential decisions include who needs closer follow-up, who is likely to progress to HFpEF, who may regress after pressure unloading, who has metabolically reversible remodeling, who should receive a metabolic intervention, and who is at risk of treatment toxicity. A metabolite panel that improves statistical prediction but does not change any of these decisions has limited clinical value. Conversely, metabolomic biomarkers that improve risk calibration and provide clinical net benefit beyond conventional factors can directly inform personalized care decisions (83).

**Figure 3:**
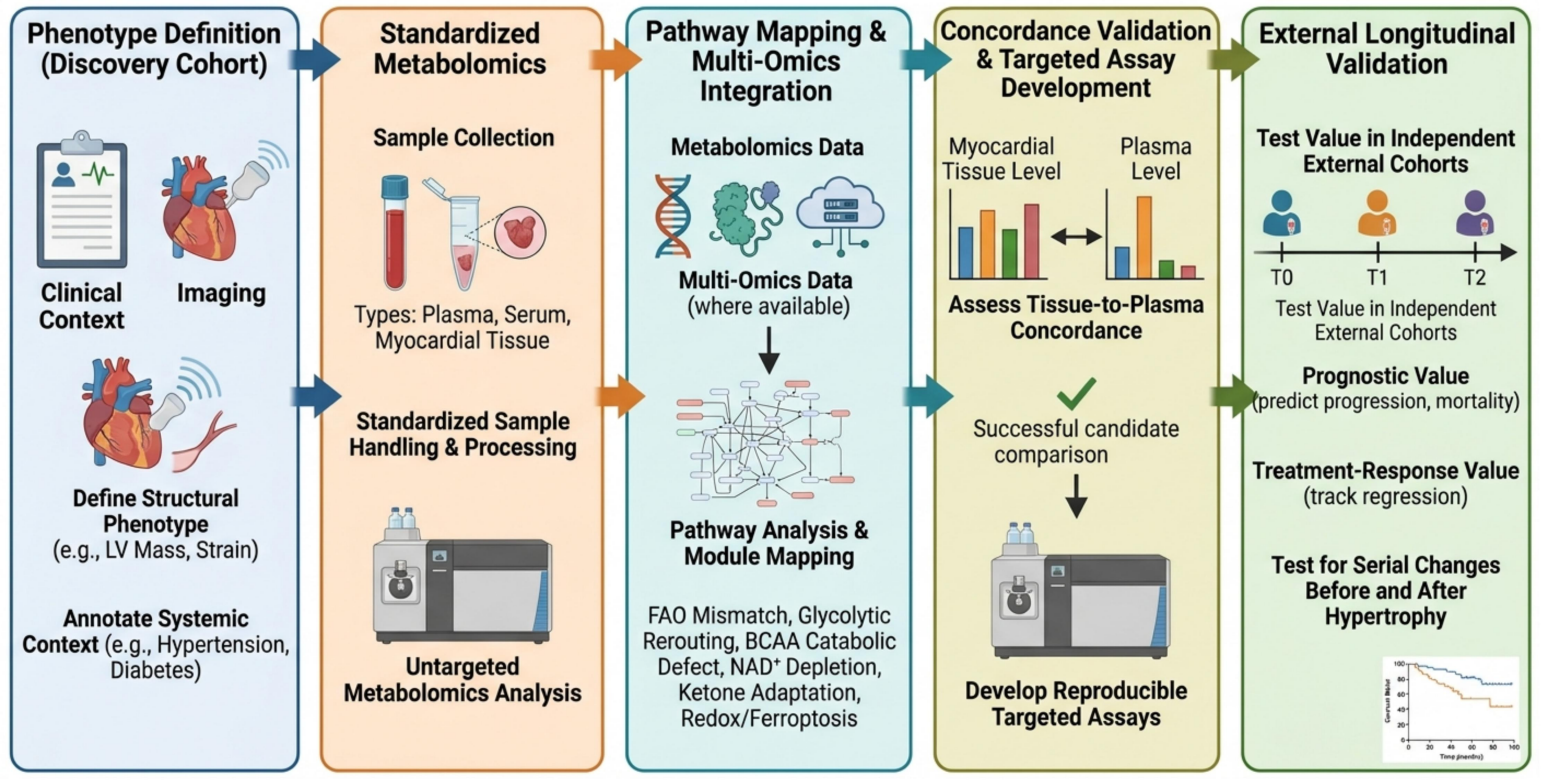
Biomarker discovery pipeline for metabolic abnormalities in myocardial hypertrophy. A proposed workflow begins with phenotype definition by imaging and clinical context, proceeds through standardized blood and tissue metabolomics, maps metabolites to pathway modules, integrates transcriptomic/proteomic data where available, validates tissue-to-plasma concordance, and tests prognostic or treatment-response value in external longitudinal cohorts.

The pathway to decision support should be staged. Discovery cohorts can use broad untargeted metabolomics, proteomics, transcriptomics, and imaging. Integrating multi-omics layers, such as genomics, metabolomics, and proteomics, can offer complementary risk information and improve predictive performance beyond single-modality approaches (70). Candidate modules should then be reduced to targeted assays that are affordable, reproducible, and interpretable. External validation should test calibration across centers, platforms, sex, ancestry, diabetes status, kidney function, and medication exposure. Finally, interventional studies should ask whether using the biomarker changes management and improves outcomes. This last step is often missing. Without biomarker-guided intervention, metabolomic research may remain descriptive even when biologically sophisticated. This discovery-to-decision pathway is shown in Figure 3.

Machine learning may help integrate metabolite panels with imaging and clinical data, but it should not obscure biology. Explainable AI techniques can map metabolic signatures to biological pathways, enabling biologically interpretable risk stratification (84). Models should report calibration, missing-data handling, feature stability, and performance in external cohorts. They should also identify whether metabolites add information beyond simple clinical variables such as blood pressure, body mass index, diabetes, renal function, natriuretic peptides, troponin, and LV mass. If a complex model performs only marginally better than standard clinical assessment, its implementation burden may not be justified.

### 5.5 Experimental model standards

Animal and cellular models remain essential, but model choice should match the clinical question. Transverse aortic constriction is powerful for pressure overload but does not represent obesity-related HFpEF. High-fat feeding combined with TAC did not exacerbate cardiac outcomes, suggesting this model may not fully capture the cardiometabolic complexity of human HFpEF (85). High-fat diet or diabetic models capture cardiometabolic stress but may not reproduce human multimorbidity. Young male mice may miss sex, age, and hormonal influences. Isolated cardiomyocytes can reveal cell-autonomous metabolic mechanisms but omit endothelial, fibroblast, immune, neural, and systemic substrate supply. Organoids and engineered tissues can improve control, but they still require validation against human myocardium.

Metabolic studies should report not only cardiac mass and function but also substrate availability, feeding state, sex, age, strain, temperature, anesthesia, and hemodynamic load. Standardized reporting of biological context, including anesthesia and hemodynamic status, is essential for reproducibility and cross-study comparison in mammalian metabolomics (86). They should distinguish myocardial tissue metabolites from plasma metabolites and should avoid inferring flux from abundance alone. Stable isotope tracing, oxygen consumption, enzyme activity, mitochondrial respiration, and tissue imaging can clarify whether a metabolite change reflects altered production, utilization, transport, or clearance. This is especially important for pathways such as FAO, glycolysis, BCAA catabolism, and ketone use, where abundance can move in opposite directions depending on flux.

The strongest translational studies will connect experimental and human findings. For example, if a mouse study identifies a BCAA catabolic defect that worsens hypertrophy, human cohorts should test whether BCAA-related metabolites associate with LV mass, fibrosis, or HFpEF outcomes, and whether the signal is independent of diabetes and kidney function. In dilated cardiomyopathy patients, cardiac BCAA accumulation was linked to blunted insulin signaling, highlighting the need to adjust for diabetic status when interpreting BCAA signals (87). If a NAD + intervention improves hypertrophy in animals, human trials should measure NAD-related metabolites, mitochondrial readouts, and structural remodeling. This bidirectional loop can prevent overinterpretation of isolated animal mechanisms.

### 5.6 Practical reporting recommendations

Future studies should report enough clinical and analytic detail to let readers decide whether metabolic signals are interpretable. Inadequate reporting of quality control procedures may lead to misinterpretation and prevent future meta-analysis of metabolomic findings (88). At minimum, human cohorts should provide hypertrophy definition, imaging modality, fasting state, diabetes status, renal function, body mass index, sex, age, medications, diet or supplement information when available, and timing of blood sampling relative to exercise, acute illness, and therapy changes. Studies should also specify whether samples were collected before or after development of hypertrophy, because prediction and phenotyping are different goals. A baseline marker that predicts future LV mass may not be the same as a marker that tracks regression after treatment.

Studies should also distinguish among biomarker categories. Diagnostic markers identify existing hypertrophy or metabolic phenotype. A multiple-marker approach integrating traditional and novel cardiac biomarkers enhances early detection of cardiovascular diseases (68). Prognostic markers predict progression, heart failure, arrhythmia, or mortality. Pharmacodynamic markers show that an intervention engaged a pathway. Surrogate response markers should change in a way that predicts clinical benefit. Many current studies blend these purposes, which makes translation difficult. For example, an acylcarnitine signature may diagnose FAO mismatch, but it should not be called a treatment-response marker unless serial changes after therapy predict improved structure or function.

Finally, metabolic biomarkers should be interpreted with humility. Myocardial hypertrophy is not a single disease and metabolism is not a one-direction pathway. As cardiovascular diseases affect multiple pathophysiological pathways, a single biomarker approach cannot be considered ideal for optimal clinical application (11). A metabolite can be a fuel, a stress marker, a signaling molecule, or a clearance product. The strongest future work will integrate chemistry, physiology, imaging, and outcomes rather than relying on isolated abundance changes. This standard is demanding, but it is necessary if metabolic abnormalities are to become actionable biomarkers rather than descriptive features of diseased myocardium.

A final translational consideration is reversibility. The most useful metabolic biomarker may not be the one most strongly associated with established hypertrophy, but the one that identifies myocardium still capable of recovery. Less than 50% of patients respond favorably to most therapies, and reversibility is influenced by age, sex, BMI, and disease aetiology (3). Fibrosis, capillary rarefaction, mitochondrial DNA damage, and chronic inflammation may reduce reversibility even if metabolic stress can be detected. Conversely, early substrate inflexibility, NAD^+^ depletion, or BCAA catabolic impairment may be modifiable before structural remodeling becomes fixed. Future cohorts should therefore include regression endpoints after blood pressure control, weight loss, valve intervention, SGLT2 inhibitor therapy, exercise training, or other targeted interventions. A marker that predicts regression could be more clinically valuable than a marker that simply tracks disease severity.

Clinical adoption will also require simplicity. Large untargeted metabolomic panels are useful for discovery, but routine cardiology practice is more likely to use small targeted panels linked to clear interpretation. Targeted and untargeted metabolomics offer complementary values, with targeted approaches being particularly suited for quantitative clinical applications (67). A practical report might classify patients into FAO-lipotoxic, glycolytic-redox, BCAA-catabolic, NAD-mitochondrial, ketone-adaptive, or ferroptosis-redox patterns, each with confidence scores and recommended validation steps. Such reporting should be tested prospectively and should remain transparent enough for clinicians to understand why a pathway label was assigned.

The same principle applies to therapeutic monitoring. A patient should not be labeled as improved simply because one metabolite moved toward a population mean. Improvement should require a coherent pattern: pathway marker change, stable renal and nutritional context, parallel improvement in imaging or function, and absence of compensatory harm in another metabolic axis. The efficacy of metabolic therapies depends critically on HF phenotype, disease stage, and global metabolic network integrity (75). This prevents false reassurance and supports more precise intervention.

## 6 Conclusions

Metabolic abnormalities in myocardial hypertrophy extend far beyond ATP shortage. They rewire substrate use, biosynthesis, redox state, mitochondrial quality control, amino acid handling, ketone adaptation, epigenetic regulation, inflammation, and inter-organ communication. The field has moved from descriptive fuel switching to mechanistic circuits that can be measured and potentially targeted. For Biomarker Research, the most promising direction is not a single universal metabolite but a phenotype-aware, pathway-anchored biomarker strategy that integrates circulating metabolites with imaging, clinical context, and validated outcomes. Such an approach could identify hypertrophy that is still metabolically reversible and guide earlier, more precise intervention.

## Glossary

ACC2: acetyl CoA carboxylase 2
AMPK: AMP-activated protein kinase
BCAA: branched-chain amino acid
BCAT: Branched-chain amino acid transaminase
BCKDH: Branched-chain α-keto acid dehydrogenase
BCKDK: Branched-chain α-keto acid dehydrogenase kinase
CAV1: caveolin 1
CD36: cluster of differentiation 36
Decr1: 2,4-dienoyl-CoA reductase 1
DPP-4: dipeptidyl peptidase 4
ERR: estrogen-related receptor
FAO: fatty acid oxidation
HFpEF: heart failure with preserved ejection fraction
IP3R: inositol 1,4,5-trisphosphate receptor
KLF7: Kruppel-like factor 7
MICU3: mitochondrial calcium uptake 3
mTOR: mechanistic target of rapamycin
NAD+: nicotinamide adenine dinucleotide
NCBI: National Center for Biotechnology Information
NRF2: nuclear factor erythroid 2-related factor 2
PFKL: phosphofructokinase, liver type
P4HB: protein disulfide-isomerase
PKA: protein kinase A
SGLT2: sodium-glucose cotransporter 2
SIRT: sirtuin
STAT3: signal transducer and activator of transcription 3
YAP: yes-associated protein
